# Computational Characterization of Astrophysical Species: The Case of Noble Gas Hydride Cations

**DOI:** 10.3389/fchem.2021.664693

**Published:** 2021-05-11

**Authors:** María Judit Montes de Oca-Estévez, Rita Prosmiti

**Affiliations:** ^1^Department of Atomic, Molecular and Surface Processes (PAMS), Institute of Fundamental Physics (IFF-CSIC), CSIC, Madrid, Spain; ^2^Atelgraphics S.L., Madrid, Spain

**Keywords:** electronic structure calculations, intermolecular potentials, molecular spectroscopic constants, computational vibrational spectroscopy, computational chemistry, quantum astrochemistry, noble gas hydride cations

## Abstract

Theoretical–computational studies together with recent astronomical observations have shown that under extreme conditions in the interstellar medium (ISM), complexes of noble gases may be formed. Such observations have generated a wide range of possibilities. In order to identify new species containing such atoms, the present study gathers spectroscopic data for noble gas hydride cations, NgH^+^ (Ng = He, Ne, Ar) from high-level *ab initio* quantum chemistry computations, aiming to contribute in understanding the chemical bonding and electron sharing in these systems. The interaction potentials are obtained from CCSD(T)/CBS and MRCI+Q calculations using large basis sets, and then employed to compute vibrational levels and molecular spectroscopic constants for all known stable isotopologues of ground state NgH^+^ cations. Comparisons with previously reported values available are discussed, indicating that the present data could serve as a benchmark for future studies on these systems and on higher-order cationic noble gas hydrides of astrophysical interest.

## 1. Introduction

Given the extreme conditions of temperature and pressure, the interstellar medium (ISM) is a perfect framework to find unconventional molecules that are unthinkable in the Earth's atmosphere. Such example is the noble gas (Ng) hydride cations, NgH^+^ (Fortenberry, 2017, 2019; Bovino and Galli, 2019; Novotný et al., 2019). The existence of these noble gas compounds is fascinating, as they are constituted by noble gases that are characterized by their high electronic stability, and in addition they are charged species with a long enough half-life time to be studied. During several decades, it was thought that noble gas compounds were quite unlikely, as they would react with other elements or molecular systems through weak van der Waals (vdW) interactions. Such proton-noble gas molecules are produced primarily in the ISM and the planetary ionospheric, and they are critical to understand and model the scenario of the early universe (Zygelman et al., 1998; Ferriére, 2001; Lepp et al., 2002).

The presence of the HeH^+^ and ArH^+^ molecules in ISM has been recently reported for first time in the planetary nebula NGC 7027 (Güsten et al., 2019) and Crab Nebula (Barlow et al., 2013), respectively, while later on, the ArH^+^ has been also detected in extragalactic sources (Müller et al., 2015), and it has been suggested that is ubiquitous in the ISM (Schilke et al., 2014). The first of these cations to be detected was argonium, ArH^+^. The Ar atom is the 11th most abundant element in the universe, with ^36^Ar and ^38^Ar being the richest isotopes of 84.6% (Lodders, 2007), in contrast to terrestrial Ar consists mostly (99.6%) of ^40^Ar. The high ionization potential is responsible for the atomic argon being neutral in the ISM (Sofia and Jenkins, 2009), but its proton and hydrogen affinity combined with possibility that cosmic rays ionize it (Barlow et al., 2013; Cueto et al., 2014; Roueff et al., 2014; Schilke et al., 2014), allows the formation of molecular species, such as ArH^+^. The discovery of this complex in the Crab nebula (Barlow et al., 2013) has been the precursor to find other noble gas molecules in the ISM.

The study of ArH^+^ complex started in 1970, when for first time, the potential energy curve (PEC) of its fundamental electronic state have been reported (Roach and Kuntz, 1970), while few years later the corresponding spectroscopic constants have been calculated (Rosmus, 1979), allowing to determine the zone of the electromagnetic spectrum in which to search for the ArH^+^ bands. Later on, the rovibrational bands of the fundamental state of ArH^+^ in the infrared (IR) spectrum have been assigned using experimentally synthesized molecules (Brault and Davis, 1982), providing its spectroscopic constants. More recently, several theoretical studies have been reported including *ab initio* or spectroscopic-derived potential curves of its ground state up to dissociation (Hirst et al., 1992; Coxon and Hajigeorgiou, 2016), as well as electronically excited states for investigating its photodissociation processes (Stolyarov and Child, 2005; Alekseyev et al., 2007; Abdoulanziz et al., 2018).

The interest in the HeH^+^, discovered in the laboratory in 1925 (Hogness and Lunn, 1925), is due to its composition, and it is made up of the two most abundant elements in the universe: helium and hydrogen. The HeH^+^ presence in the ISM has been speculated for a long time, and its detection has been just recently confirmed (Güsten et al., 2019). Moreover, this cation is also the simplest molecular system to treat just isoelectronic with H_2_, so it has been extensively studied theoretically and experimentally ever since (Wolniewicz, 1965; Kolos, 1976; Kolos and Peek, 1976; Bishop and Cheung, 1979; Bernath and Amano, 1982; Carrington et al., 1983; Crofton et al., 1989; Cencek et al., 1995; Juřek et al., 1995; Liu and Davies, 1997; Matsushima et al., 1997; Coxon and Hajigeorgiou, 1999; Engel et al., 2005; Stanke et al., 2006; Pachucki, 2012; Tung et al., 2012; Perry et al., 2014), including observation of infrared and rotational spectra of its isotopologues, *ab initio* and high accurate with spectroscopic precision potential curves, and quasibound states calculations.

With respect to the heavier noble gases, Ne is the fifth most abundant element in the universe, while Kr is so rare, and Xe is rarer still in the ISM (Rogers et al., 1987; Gruet and Pirali, 2019; Grandinetti, 2020). Although natural compounds such as NeH^+^ have not yet been found, the confirmed presence of Ne in the moon's exosphere (Benna et al., 2015) allows to speculate with the existence NeH^+^ in cold planetary systems, where the interaction of Ne with H or H^+^ is possible. If this or other Ne-containing molecules are discovered, the evolution of our solar system could be better understood. Therefore, earlier and recent studies on such interactions have been reported from *ab initio* calculations and the interplay between theory and experiment has also been investigated (Gianturco et al., 1987; Hirst et al., 1992; Civiš et al., 2004; Gerivani et al., 2015; Coxon and Hajigeorgiou, 2016).

Thus, the purpose of this research is to provide potential curves obtained from benchmark *ab initio* electronic structure calculations for HeH^+^, NeH^+^, and ArH^+^, which would be capable of accurate predictions of energetics and spectroscopic properties for all their isotopologues aiming to facilitate the astrochemical detection of such noble gas compounds in new ISM regions. Utilization of quantum chemical tools to analyze and characterize these cations has become essential to provide accurate rovibrational spectroscopic constants to assist telescopic observations and laboratory experiments (Fortenberry, 2017). In order to assign the observed signals of the rotational and vibrational spectra, it is necessary to have information about the spectrum of the possible detected molecules. To do so, experiments in the laboratory have to be carried out in which the spectrum of these molecules is obtained. Nowadays, for neutral and closed-shell molecules in gas phase it is quite easy to create them in the laboratory and to obtain their spectrum, even at very low pressure to simulate the conditions of the ISM. However, the molecules that are detected in the space can show different behavior compared with the ones that are found in the Earth as these molecules are under extreme conditions in the universe. Therefore, it might be difficult to create them in a laboratory. For example, charged and radical species are difficult to create because of their high reactivity. Even if they are synthesized, their short lifetimes makes almost impossible to obtain the vibrational and rotational spectrum. That experimental shortcoming is where computational chemistry comes into play, especially data-driven quantum chemistry approaches on structural, spectroscopic, and electronic properties. This branch of computational chemistry has the potential to improve the decision making in astrophysics and astrochemistry projects. In this way, the search of new molecular species that are not stable in the laboratory or that are difficult to synthesize (for example radicals and charged isolated molecules) could be significantly simplified.

## 2. Computational Details

All *ab initio* electronic structure calculations were carried out using Molpro program (Werner et al., 2012). The DENEB software package den (2020) was employed to generate and organize all input and output data files, respectively. As a first step, the geometries of each system at its ground X^1^Σ^+^ electronic state were optimized at CCSD(T)/aug-cc-pV6Z level of theory together with the corresponding harmonic frequency analysis. In turn, we employed different levels of theory, such as the second-order Möller-Plesset perturbation (MP2), coupled cluster single-double and perturbative triple excitations [CCSD(T)], the explicit correlated CCSD(T)-F12, correlating all valence electrons of the noble gas atoms, as well as the multireference configuration interaction, with singles and doubles, including Davidson correction (MRCI+Q) methods, to compute total and interaction energies of these molecules by varying the diatomic R (Ng–H^+^) bondlengths. The MRCI reference wave function was of the complete active space self-consistent field (CASSCF) calculations with the full valence active space of 8 electrons distributed on 10 orbitals (6a_1_, 2b_1_, 2b_2_) for NeH^+^ and 11 (6a_1_, 2b_1_, 2b_2_, 1a_2_) for ArH^+^. The inner 1s orbital of Ne and the 1s, 2s, and 2p of Ar were kept doubly occupied in all configurations. The NgH^+^ are closed-shell systems, and thus spin-orbit splitting effects are neglected in our ground state calculations.

For all configurations studied, the basis set superposition error (BSSE) was corrected by the counterpoise method (Boys and Bernardi, 1970), with the interaction between Ng and H^+^ computed as $\Delta E=E_{NgH^{+}}-E_{Ng}-\delta E_{BSSE}$. Further, different correlation-consistent basis sets, such as valence-only aug-cc-pV*n*Z (AV*n*Z), as well as the core-valence aug-cc-pCV*n*Z (ACV*n*Z), with *n* = Q, 5 and 6, and the weighted aug-cc-pwCV*n*XZ (AwCV*n*Z) sets with *n* = Q and 5 for the Ng atoms were employed (Werner et al., 2012). Supplementary Figure 1 (see Supplementary Material) depicts the convergence of total and interactions CCSD(T) energies using the core-valence ACV*n*Z and AwCV*n*Z sets to the CBS[56] estimate from the valence-only calculations (Schwartz, 1962). On the basis of these convergence tests, we consider the AV*n*Z basis sets, hereafter, and the extrapolation of the computed CCSD(T) energies at their (approximate) complete basis set (CBS) limit were carried out by checking various extrapolation schemes, such as the mixed Gaussian/exponential form (Peterson et al., 1994), $E_{n}=E_{CBS}+Ae^{-\left(n-1\right)}+Be^{-{\left(n-1\right)}^{2}}$ for the total energies, and the two-parameter expression (Schwartz, 1962) given by $E_{n}=E_{CBS}+A/n^{3}$, where *n* is the cardinal number, applied to the correlation energies, with the extrapolated correlation energies added then to the AV6Z Hartree-Fock energies. The extrapolation was performed for all individual energies, and the CP corrected interaction energies were computed thereafter. In this way, energy estimates were obtained at the CCSD(T)/CBS[56] level of theory, that is often referred to as the gold standard of quantum chemistry (Ramabhadran and Raghavachari, 2013) for single reference wavefunction molecular systems. Further, dipole moment expectation values are also computed as a function of *R* from all electron CCSD/AV6Z calculations including the relaxation contribution (Werner et al., 2012).

In order to spectroscopically characterize these noble gas cations and their more abundant isotopologues, it is necessary to obtain an accurate PEC. To do this, the computed PECs are compared with the most recent theoretical and experimental ones available. In total, 200 points along *R* distances between 0.3 and 30 Å were used to build the PEC for each molecule by combining cubic interpolation splines with long-range term for *R* ≥ 10 Å, in order to provide information on energetics and formation mechanisms. Vibrational states were calculated by solving numerically (using the Numerov–Cooley algorithm) the one-dimensional (radial) time-independent Schrödinger equation with the diatomic Hamiltonian being $H=-\frac{\hbar^{2}}{2\mu }\frac{\partial^{2}}{\partial R^{2}}+\frac{J\left(J+1\right)}{2\mu R^{2}}+V\left(R\right)$, where *V*(*R*) are the calculated Ng–H^+^ PECs, with *R* being the distance between the two atoms, μ is the reduced mass of the system given by $\frac{1}{\mu }=\frac{1}{m_{Ng}}+\frac{1}{m_{H^{+}}}$, where *m*_*Ng*_ and $m_{H^{+}}$ are the atomic masses of ^3,4^He, ^20,21,22^Ne, ^36,38,40^Ar, and H or D isotopes (Coursey et al., 2005), respectively, and *J* the rotational quantum number, being zero for pure vibrational states. Molecular and spectroscopic constants are estimated for all above-mentioned isotopologues. The vibrational constants ω_*e*_ and ω_*e*_x_*e*_ were obtained by fitting the vibrational energies E(υ) to the expression: $E\left(\upsilon \right)=\omega_{e}\left(\upsilon +1/2\right)-\omega_{e}x_{e}{\left(\upsilon +1/2\right)}^{2}$, while the rotational constants are given by ${\text{B}}_{e}=\frac{1}{2\mu R_{e}^{2}}$, with *R*_*e*_ being the equilibrium bond length, and B_υ_ as the expectation value $\langle \chi_{\upsilon }|\frac{1}{2\mu R^{2}}|\chi_{\upsilon }\rangle$, with χ_υ_ the corresponding vibrational eigenfunction.

## 3. Results and Discussion

### 3.1. *Ab initio* Electronic Structure Calculations and PECs

**Figure 1:**
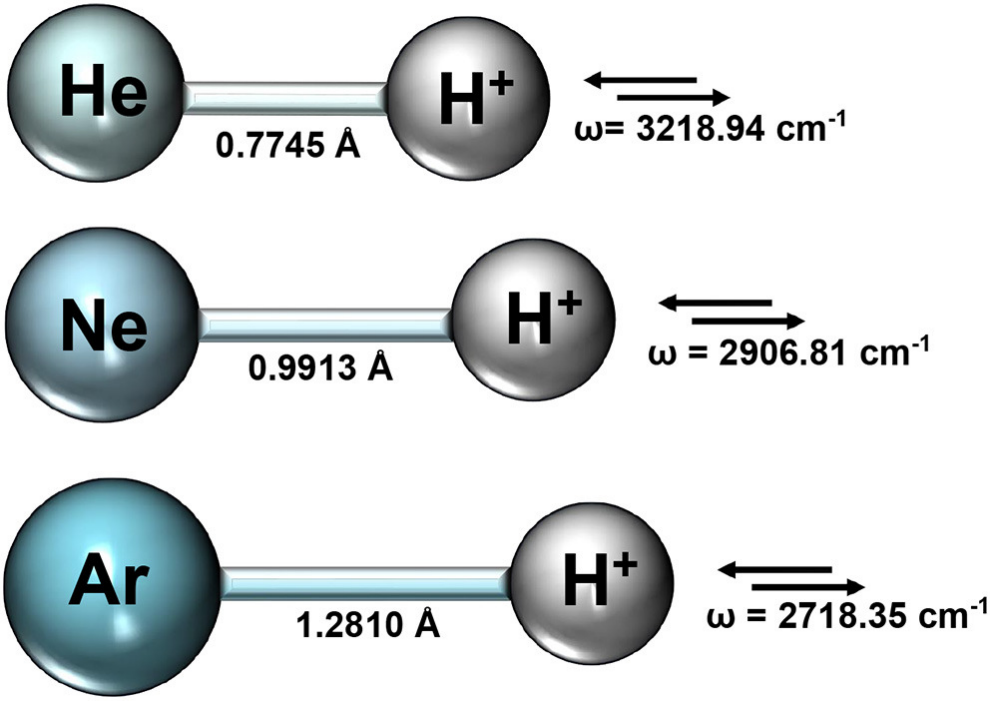
Equilibrium bondlengths, *R*_*e*_, and vibrational mode harmonic frequencies from the CCSD(T)/AV6Z optimization calculations for each NgH^+^ molecule.

We first performed optimization calculations for each NgH^+^ molecule at CCSD(T)/AV6Z level of theory, and the obtained equilibrium bondlengths *R*_*e*_ and vibrational stretching mode harmonic frequencies are summarized in Figure 1.

The rather short bondlengths range from 0.774461, 0.991304, and 1.281013 Å, and the exceptionally high vibrational frequencies between 3219 and 2718 cm^−1^ going from HeH^+^ to ArH^+^, respectively, indicate the clearly dominant covalency of these molecules. Thus, by considering the equilibrium geometries from the above CCSD(T)/AV6Z optimizations, we carried out single point calculations at different levels of theory, such as MP2, CCSD(T), CCSD(T)-F12, and MRCI+Q, employing large AVXZ (X = Q, 5, 6) basis sets, and then extrapolations to CCSD(T)/CBS limit considering both two- and three-step schemes (Schwartz, 1962; Peterson et al., 1994). The computed total energies at equilibrium distances are listed in Table 1 and compared with the most accurate results reported in the literature for each molecule. We should note that the most accurate value reported up to date for the HeH^+^ has a precision of 10^−12^ a.u. (Pachucki, 2012). One can see that the energies of the present CCSD(T) calculations using the largest AV6Z basis are higher than those previously available for the HeH^+^, while energies are much lower for the NeH^+^ and ArH^+^ molecules in comparison with earlier data. As can be seen, the MRCI+Q/AV6Z energies are close to those from the CCSD(T) computations, while the CCSD(T)/CBS[56] extrapolation approach provides the lower estimates compared to the best available values in the literature for all systems under study. In particular, for the HeH^+^ the CCSD(T)/CBS[56] total energy value is predicted by 3.6 cm^−1^ lower than best known (“exact”) value in the literature (Pachucki, 2012).

**Table 1:** Comparison of total energy values (in a.u.) at equilibrium distance of R obtained in the present work from the indicated calculations and the best estimates of previous studies.

	**HeH^**+**^**	**NeH^**+**^**	**ArH^**+**^**
Literature	−2.9786667^*a*^	−128.902477^*f*^	−527.179256^*f*^
	−2.97868906^*b*^	−128.944424^*g*^	−527.210000^*g*^
	−2.97870262^*c*^	−128.943538^*h*^	−527.204368^*i*^
	−2.978706591^*d*^		
	−2.978708310771^*e*^		
MP2/AVQZ	−2.97119612	−128.92535770	−527.19676773
MP2/AV5Z	−2.97212128	−128.93866766	−527.20640683
MP2/AV6Z	−2.97249327	−128.94360982	−527.21174138
CCSD(T)/AVQZ	−2.97753886	−128.93261021	−527.22479619
CCSD(T)/AV5Z	−2.97822081	−128.94453910	−527.23319916
CCSD(T)/AV6Z	−2.97846538	−128.94853590	−527.23725238
CCSD(T)−F12/AVQZ	−2.97854415	−128.94837833	−527.22436255
MRCI+Q/AV6Z	−2.97846538	−128.94851552	−527.24011465
CCSD(T)/CBS[Q56]	−2.97860770	−128.95086138	−527.23961265
CCSD(T)/CBS[56]	−2.97872479	−128.95363280	−527.24247792

a *Wolniewicz (1965),*

b *Kolos and Peek (1976),*

c *Bishop and Cheung (1979),*

d *Cencek et al. (1995),*

e *Pachucki (2012),*

f *Rosmus (1979),*

g *Hirst et al. (1992),*

h *Pendergast et al. (1994),*

i *Stolyarov and Child (2005)*.

**Figure 2:**
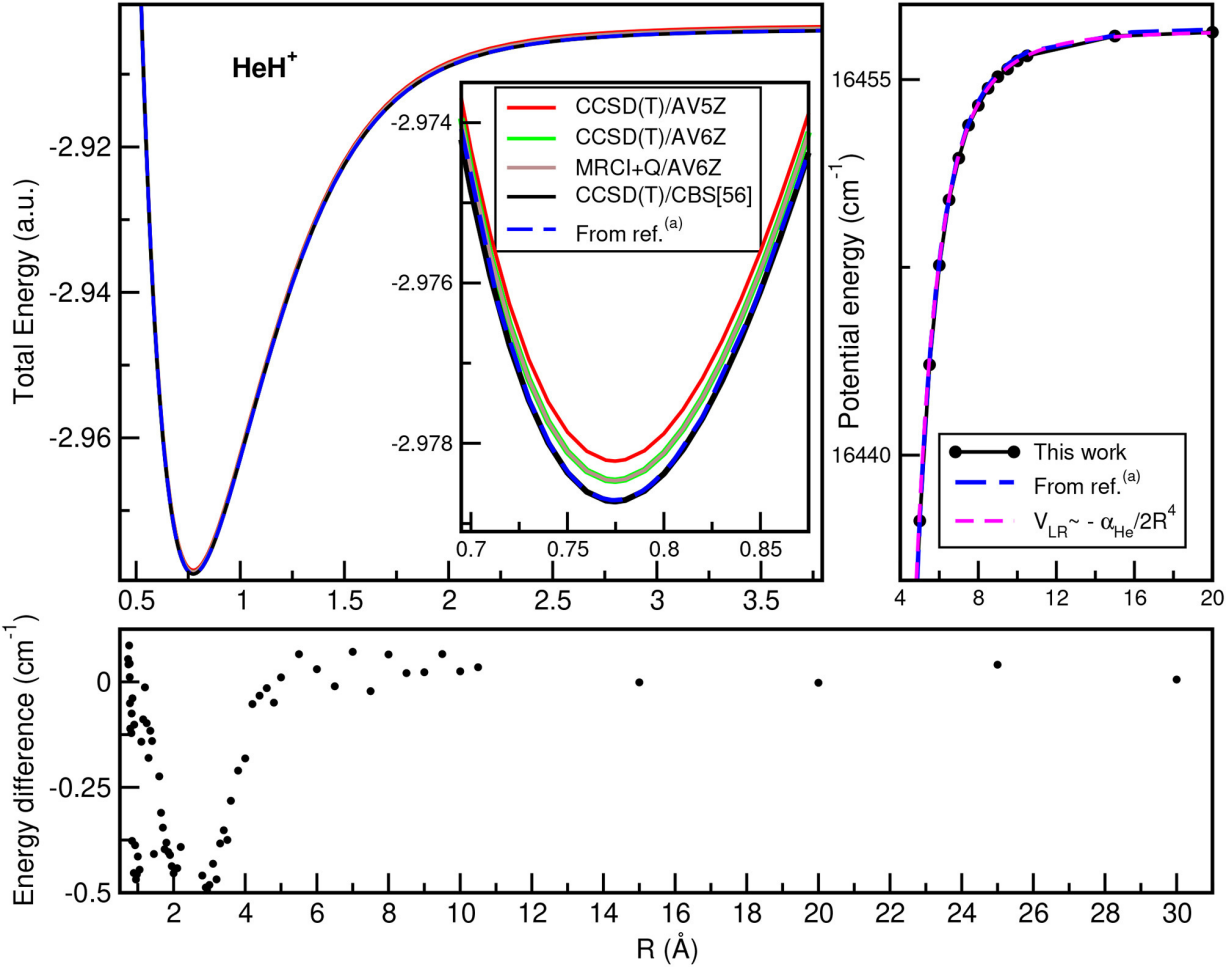
Total **(upper left)** and potential **(upper right)** energies of HeH^+^ molecule as a function of *R* bondlength at the indicated levels of theory, and their comparisons at long-range region (see text). The energy differences between the present CCSD(T)/CBS[56] data with the best known values from ^(*a*)^Pachucki (2012) are also displayed **(lower)**.

**Figure 3:**
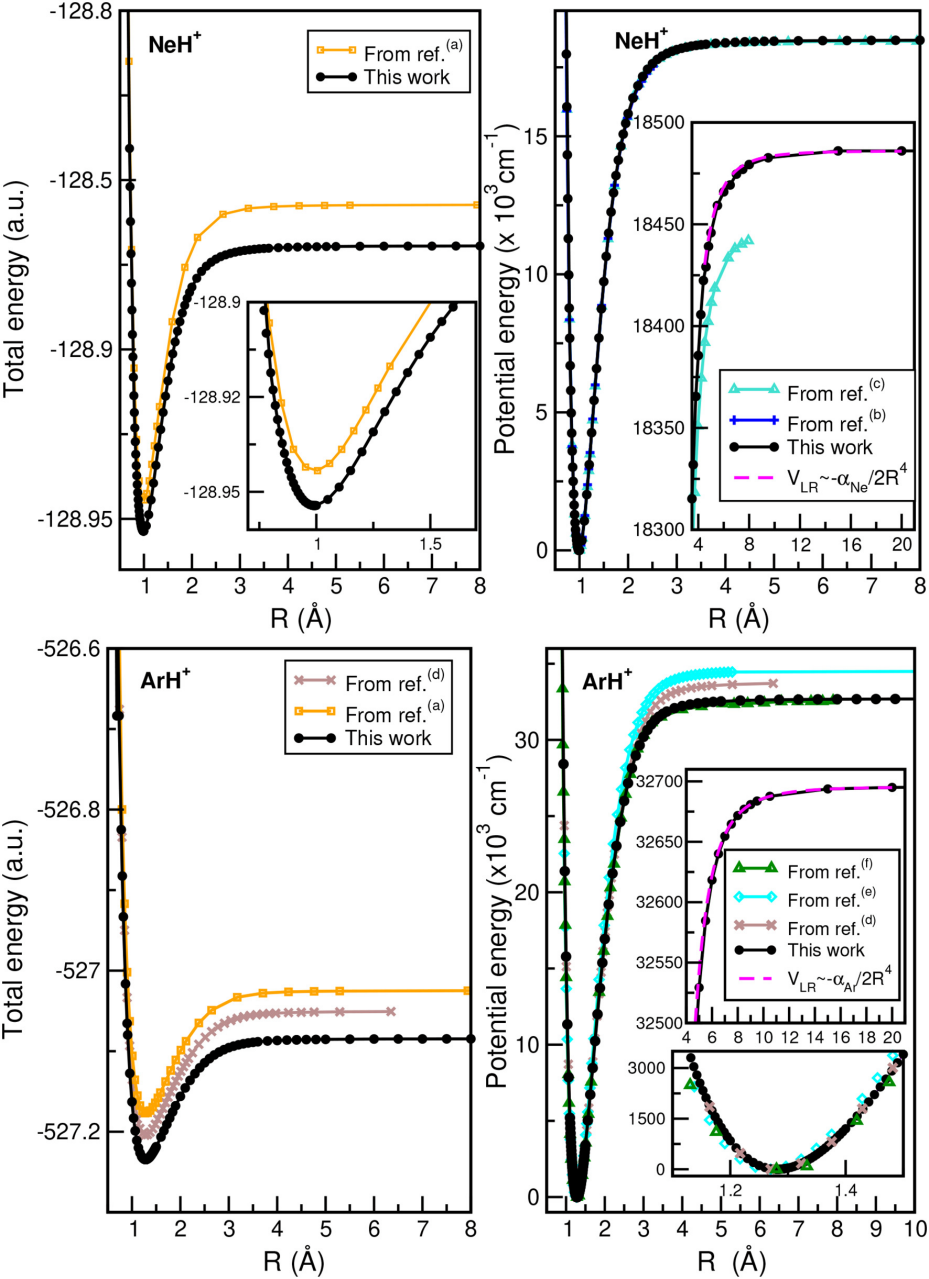
Total **(left)** and potential **(right)** energies of NeH^+^ **(upper)** and ArH^+^ **(lower)** molecules as a function of *R* bondlength obtained from the present CCSD(T)/CBS[56] calculations, and comparison with data available in previous studies from ^(*a*)^Hirst et al. (1992), ^(*b*)^Coxon and Hajigeorgiou (2016), ^(*c*)^Gerivani et al. (2015), ^(*d*)^Stolyarov and Child (2005), ^(*e*)^Alekseyev et al. (2007), and ^(*f*)^Abdoulanziz et al. (2018).

In Figure 2, we plot the total (see upper left panel) and potential (see upper right panel) energies obtained from the present CCSD(T)/CBS[56] calculations as a function of the He–H^+^ distance, in comparison with CCSD(T) and MRCI+Q data, as well as their differences (see lower panel) with the most accurate values available (Pachucki, 2012) along *R*, while Figure 3 presents total and potential CCSD(T)/CBS[56] energies from the present work in comparison with values of previous studies (Hirst et al., 1992; Stolyarov and Child, 2005; Alekseyev et al., 2007; Gerivani et al., 2015; Coxon and Hajigeorgiou, 2016) as a function of the Ne–H^+^ (see upper panels) and Ar–H^+^ (see lower panels) distances.

In the HeH^+^ case, the CCSD(T)/CBS[56] potential energies show very small differences with the high accurate Born-Oppenheimer potential reported by (Pachucki, 2012) (see lower panel of Figure 2), while in the cases of NeH^+^ and ArH^+^ larger differences are found with previous studies (see Figure 3), especially for the ArH^+^ potential, as we will discuss later on.

An important aspect of the potential curves, with strong influence on bound and quasi-bound states in the dissociation limit, is the correct behavior at such asymptotes. At large intermolecular distances, the ground NgH^+^(X^1^Σ^+^) states dissociate to Ng(^1^S_0_) + H^+^, with the ion-induced dipole interaction between the Ng atom and the proton being the leading long-range potential term. In Figure 2 (see upper right panel) and Figure 3 (see right panels), we display the asymptotic behavior of the interaction as obtained from the present calculations in comparison with the expected long-range attractive potential term given by V_LR_(*R*)=$-\frac{\alpha_{\text{Ng}}}{2R^{4}}$, with α_Ng_ being the dipole polarizability of He, Ne, and Ar atoms obtained from Mitroy et al. (2010). The interaction between an Ng atom with a proton increases from He to Ar, as the polarizability of the heavier atoms increases leading in this way to stronger interaction. One can see that the agreement is excellent between the calculated curves for each molecule, indicating the quality of the present electronic quantum calculations in these potential regions too.

In turn, we will also discuss the energetics of the simplest pathways for their formation given by the following gas phase reactions:

$$\begin{array}{l} \text{ Ng}+{\text{H}}^{+}\to {\text{NgH}}^{+}\;(1)\\ {\text{ Ng}}^{+}+\text{H}\to {\text{NgH}}^{+}\;(2)\\ \text{ Ng}+{\text{H}}_{2}^{+}\to {\text{NgH}}^{+}+\text{H }(3)\\ {\text{Ng}}^{+}+{\text{H}}_{2}\to {\text{NgH}}^{+}+\text{H }(4) \end{array}$$

In Table 2, the formation energies from CCSD(T)/AV5Z/AV6Z/CBS[56] calculations at T = 0 K for each of the above (1–4) reactions are listed. The results of the CCSD(T)/CBS[56] and MRCI+Q/AV6Z are in agreement within less than 1 kcal/mol, and compare well with energies available from recent studies (Tan and Kuo, 2019; Grandinetti, 2020), predicting lower energies in all cases. We should note that experimental enthalpies reported (Grandinetti, 2020) include both zero-point-energy (ZPE) and thermal corrections. As we will discuss later on, the ZPE effects count to 4.50, 4.07, 3.84, 6.23, and 3.71 kcal/mol for HeH^+^, NeH^+^, ArH^+^, H_2_, and ${\text{H}}_{2}^{+}$, respectively. By taking them into account (see values in parenthesis in Table 2), a closer agreement to the experimental data is achieved. Positive energy values will favor reactants, while contrary negative values will favor products. As can be seen all reactions are exothermic, with the exceptions of the reaction (3) for He and Ne atoms reacting with ${\text{H}}_{2}^{+}$ ground-state ion.

**Table 2:** Formation energies at T = 0 K (in kcal/mol) for Ng–H^+^ complexes at the indicated levels of theory, and their comparison with recently reported experimental/theoretical data.

**Ng**	**CCSD(T)**	**Literature**
	**AV5Z/AV6Z/CBS[56]**	
**Ng + H**^**+**^ **→ NgH**^**+**^		
**He**	−47.05/−47.05/−47.05(−42.55)	−42.5^*a*^
**Ne**	−51.51/−52.02/−52.47(−48.40)	−47.5^*a*^/−52.71^*b*^
**Ar**	−92.94/−93.28/−93.57(−89.73)	−88.2^*a*^/−93.27^*b*^
**Ng**^**+**^ **+ H → NgH**^**+**^		
**He**	−300.13/−300.25/−300.41(−295.91)	−295.9^*a*^
**Ne**	−236.53/−236.88/−237.35(−233.28)	−231.2^*a*^
**Ar**	−143.86/−144.26/−144.74(−140.90)	−138.0^*a*^
**Ng +** ${\text{H}}_{2}^{+}$ **→ NgH**^**+**^ **+ H**		
**He**	17.48/17.33/17.12(17.91)	19.6^*a*^
**Ne**	11.53/11.53/11.55(11.91)	14.6^*a*^
**Ar**	−29.12/−29.15/−29.18(−29.05)	−26.1^*a*^
**Ng**^**+**^ **+ H**_**2**_ **→ NgH**^**+**^ **+ H**		
**He**	−191.48/−191.60/−191.76(−190.03)	−191.7^*a*^
**Ne**	−127.18/−127.47/−127.80(−125.64)	−127.0^*a*^
**Ar**	−34.51/−34.84/−35.25(−32.86)	−33.8^*a*^

*Values in parenthesis include ZPE corrections*.

a *Grandinetti (2020)*,

b *Tan and Kuo (2019)*.

The computations predict that the formation of all NgH^+^ molecules is more favorable through reaction (2), then follows the reaction (4) for HeH^+^ and NeH^+^, while for ArH^+^ is the reaction (1) the second more favorable pathway. However, the most likely formation pathways will not only follow thermodynamic results but kinetic patterns. The Ng atoms are likely not going to ionize first and will require some leaving group to carry about the excess energy kinetically. Hence, mechanism (3) is the most likely pathway to occur, and the mechanism (1) follows as the second-most likely. Further, in astrophysical environments, depending on the regions of ISM that such molecules have been observed, it proves the main corresponding mechanism of their formation, and numerous investigations have been reported (Zygelman et al., 1998; Barlow et al., 2013; Bovino and Galli, 2019; Fortenberry, 2019; Forrey et al., 2020). For example, the ArH^+^ has been found in highly ionized regions, as well as in areas containing large quantity of molecular hydrogen, and its formation has been, in particular, ascribed to reactions (3) and (4) (Barlow et al., 2013; Theis and Fortenberry, 2015). In turn, the HeH^+^ observed in planetary nebulae (like NGC 7027), where He^+^ and H exist simultaneously due to flow patterns, mainly arises from the radiative association pathway (2), while at low temperature it can be formed via direct radiative processes (1), that takes place entirely on their ground electronic state potentials (Zygelman et al., 1998; Fortenberry, 2019).

**Figure 4:**
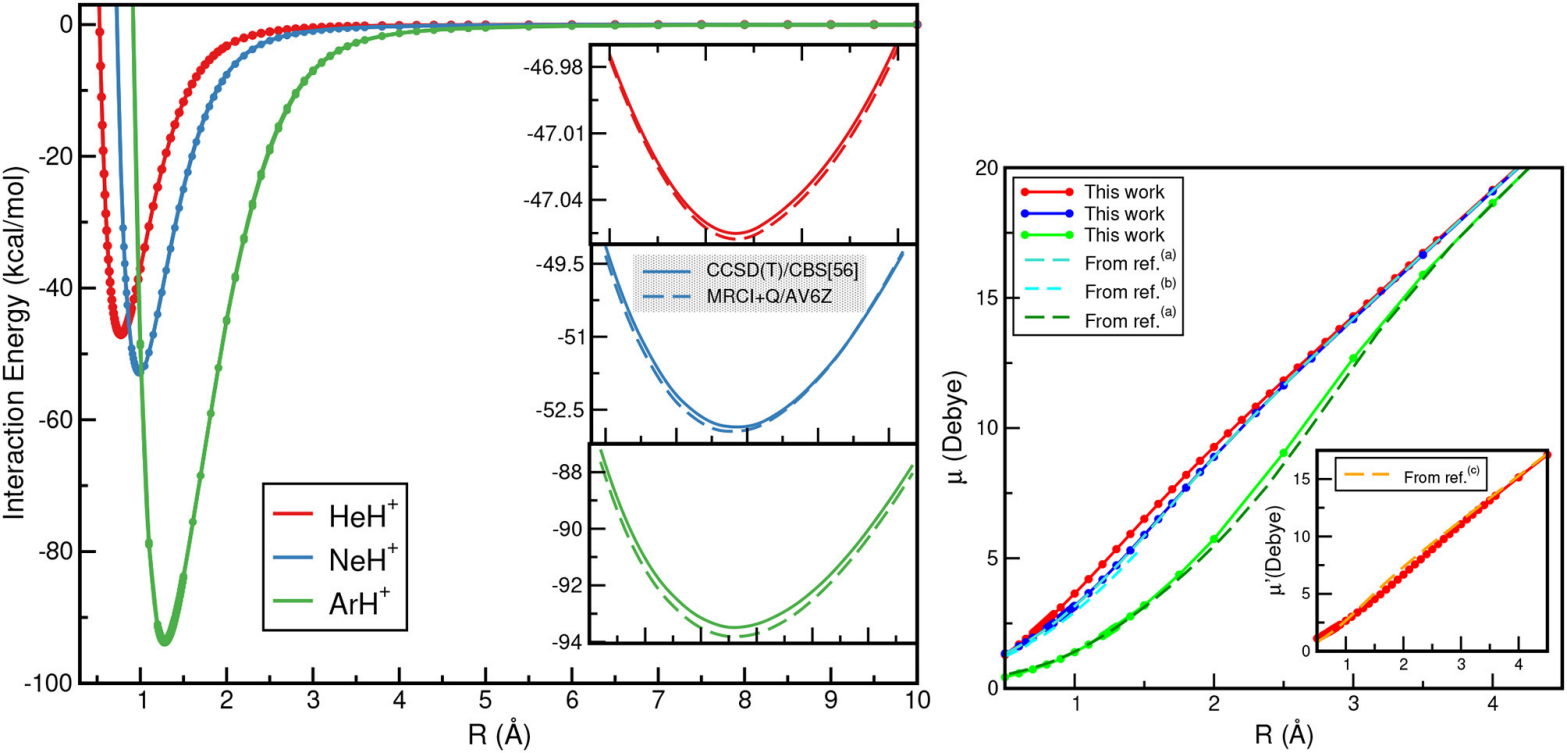
CCSD(T)/CBS[56] (solid lines) and MRCI+Q (dashed lines) interaction energies **(left)**, and dipole moment values **(right)** for the HeH^+^ (red lines), NeH^+^ (blue lines) and ArH^+^ (green lines) as a function of *R*. Electric dipole moments μ are with respect to Ng atom as origin, while μ′ with respect to the HeH^+^ center of mass are also given for comparison reasons from ^(*a*)^Hirst et al. (1992), ^(*b*)^Gerivani et al. (2015), and ^(*c*)^Juřek et al. (1995).

The calculated ground electronic state CCSD(T)/CBS[56] and MRCI+Q interaction energies as a function of *R* for each of these NgH^+^ species are displayed (see left panel) in Figure 4. The most notable aspect of them is that the depth of the potential well increases as the Ng atom becomes heavier, with those of the ArH^+^ being deeper, as expected, than its counterparts. In Figure 4 (see right panel), the dipole moments as a function of internuclear distance *R* are also plotted. The computed CCSD dipole moment expectation values μ were determined with respect to the center of mass of the molecular ion, while for comparison reasons, μ′ values for the HeH^+^ with respect to its center of mass are also calculated.

The dipole moment values for HeH^+^ and NeH^+^ show an almost linear dependence as *R* increases, while for ArH^+^ show a slightly different behavior at intermediate *R* distances. The dipole moment values calculated at the corresponding equilibrium distances of HeH^+^, NeH^+^, and ArH^+^ are 2.4804, 3.1259, and 2.3013 D, respectively, and these values agree well with the most recent theoretical and experimental values available (Hirst et al., 1992; Juřek et al., 1995; Stolyarov and Child, 2005; Gerivani et al., 2015).

In Table 3, the equilibrium distances and corresponding well depths are collected for all NgH^+^ under study, and compared with those reported previously. In particular, when the results obtained in the present work are compared with the available in the literature, the trends observed are depended on the molecule. We found that our results overestimate both R_*e*_ and D_*e*_ values compared to the experimental energies reported for each system (Ram et al., 1985; Hotop et al., 1998; Coxon and Hajigeorgiou, 1999), presenting differences of 0.0002, 0.0001, and 0.0004 Å and around of 0.7, 136, and 231 cm^−1^ for HeH^+^, NeH^+^, and ArH^+^, respectively. For HeH^+^, numerous *ab initio* potentials are available in the literature, with the most accurate “exact” theoretical value with a precision of 10^−12^ a.u. reported recently by Pachucki (2012). The present D_*e*_ value obtained from the CCSD(T)/CBS[56] and MRCI+Q calculations is within 0.15 and 0.78 cm^−1^, respectively, of this reference value, while most of previous studies show in the best case a difference of 3 cm^−1^.

**Table 3:** Equilibrium distances (R_*e*_) and well-depths (D_*e*_) for the ground NgH^+^ PECs, and comparison with theoretical as well as experimental data available.

**Method/Basis set**	***R*_*e*_(Å)**	**D_***e***_(cm^**−1**^)**	**δ(cm^**−1**^)^*a*^**
**HeH**^**+**^			
CCSD(T)/CBS[56]	0.7745	16456.95	0.71
MRCI+Q/AV6Z	0.7745	16457.88	1.64
Theory (Orville-Thomas, 1980)	0.774	16477.9	21.66
Theory (De Fazio et al., 2012)	0.7747	16460.1	3.86
Theory (Pachucki, 2012)	0.7743	16457.1	0.86
Expt. (Coxon and Hajigeorgiou, 1999)	0.7743	16456.24	-
**NeH**^**+**^			
CCSD(T)/CBS[56]	0.9913	18486.02	136.02
MRCI+Q/AV6Z	0.9913	18519.54	169.54
Theory (Rosmus, 1979)	0.9959	18357	7.0
Theory (Pendergast et al., 1994)	0.9917	18551	201.0
Theory (Civiš et al., 2004)	0.9912	18519.7	169.7
Theory (Gerivani et al., 2015)	0.9927	18449	90.0
Expt. (Ram et al., 1985; Hotop et al., 1998)	0.9912	18350	-
**ArH**^**+**^			
CCSD(T)/CBS[56]	1.2810	32695.50	231.49
MRCI+Q/AV6Z	1.2810	32811.50	347.50
Theory (Stolyarov and Child, 2005)	1.2790	33391.3	927.30
Theory (Alekseyev et al., 2007)	1.2680	34601.2	2137.20
Theory (Abdoulanziz et al., 2018)	1.2801	32576.7	112.70
Theory (Coxon and Hajigeorgiou, 2016)	1.2803	32460	-
Expt. (Hotop et al., 1998)	1.2806	32464	-

a *δ =*$D_{e}^{theor}$-$D_{e}^{expt}$.

Despite that NeH^+^ have not been observed in the ISM yet, there are various *ab initio* studies trying to characterize it and providing spectroscopic constants with the most relevant and current results on equilibrium distances and well-depths given in the early work by Rosmus (1979), later on by Pendergast et al. (1994), and from more recent CCSD(T) and MRCI calculations (Civiš et al., 2004; Gerivani et al., 2015). By comparing the present CCSD(T)/CBS[56] values, with the most recent MRCI data (Gerivani et al., 2015) differences of 0.0014 Å and 46 cm^−1^ are found in the *R*_*e*_ and D_*e*_, respectively, indicating a stronger binding in the present calculations.

Previous theoretical studies on the ground state ArH^+^ have also determined equilibrium energies and bondlengths from MP2, CCSD(T), and MRCI calculations (Hirst et al., 1992; Stolyarov and Child, 2005; Alekseyev et al., 2007; Coxon and Hajigeorgiou, 2016; Abdoulanziz et al., 2018), and in Table 3 some of them are listed. One can see that both CCSD(T)/CBS[56] and MRCI+Q values predict stronger Ar–H^+^ interaction by 119 cm^−1^ and shorter bondlength by 0.0009 Å than the most recent results published so far (Abdoulanziz et al., 2018).

### 3.2. Bound-State Calculations and Molecular Spectroscopic Constants

On the basis of the present CCSD(T)/CBS[56] PECs, vibrational bound state calculations were carried out, aiming to investigate the effect of the potential form on the vibrational states, and to further validation of the interactions by comparisons of the obtained spectroscopic constants with available experimental data taken into account ZPE effects. Given the importance of different isotopes, due to their abundance in ISM, and thus their potential detection in a variety of astrophysical environments, we decide to consider the ^3^He, ^4^He, ^20^Ne, ^21^Ne, ^22^Ne, ^36^Ar, ^38^Ar, ^40^Ar isotopes for the Ng atoms, and D, H for the hydrogen one. In Supplementary Tables 1–3 (see Supplementary Material), we list all bound vibrational energies up to their Ng + H^+^ dissociation limit.

The CCSD(T)/CBS[56] potentials support 12 (υ = 0–11) vibrational levels for the hydrogenated ^3^He/^4^HeH^+^ species in agreement with the most accurate theoretical data (Stanke et al., 2006), and 15 for the deuterated ^3^He/^4^HeD^+^ isotopomers. For the NeH^+^ and NeD^+^ systems, the present CCSD(T)/CBS[56] curve predicts 15 and 21 vibrational states, respectively, in accord with previous theoretical studies (Civiš et al., 2004; Gerivani et al., 2015), while for ArH^+^ and ArD^+^ isotopes we obtained 27 and 30 levels, respectively, that are 4 vibrational bound states more than previously reported ones for the ^40^ArH^+^ (Hirst et al., 1992; Abdoulanziz et al., 2018). This could be due to the fact of the correct asymptotic description of the curves at long-range region with R > 8 Å as shown in Figure 3, that also contributes to more bound complexes according to the calculated CCSD(T)/CBS[56] potential well depths (see Table 3).

**Figure 5:**
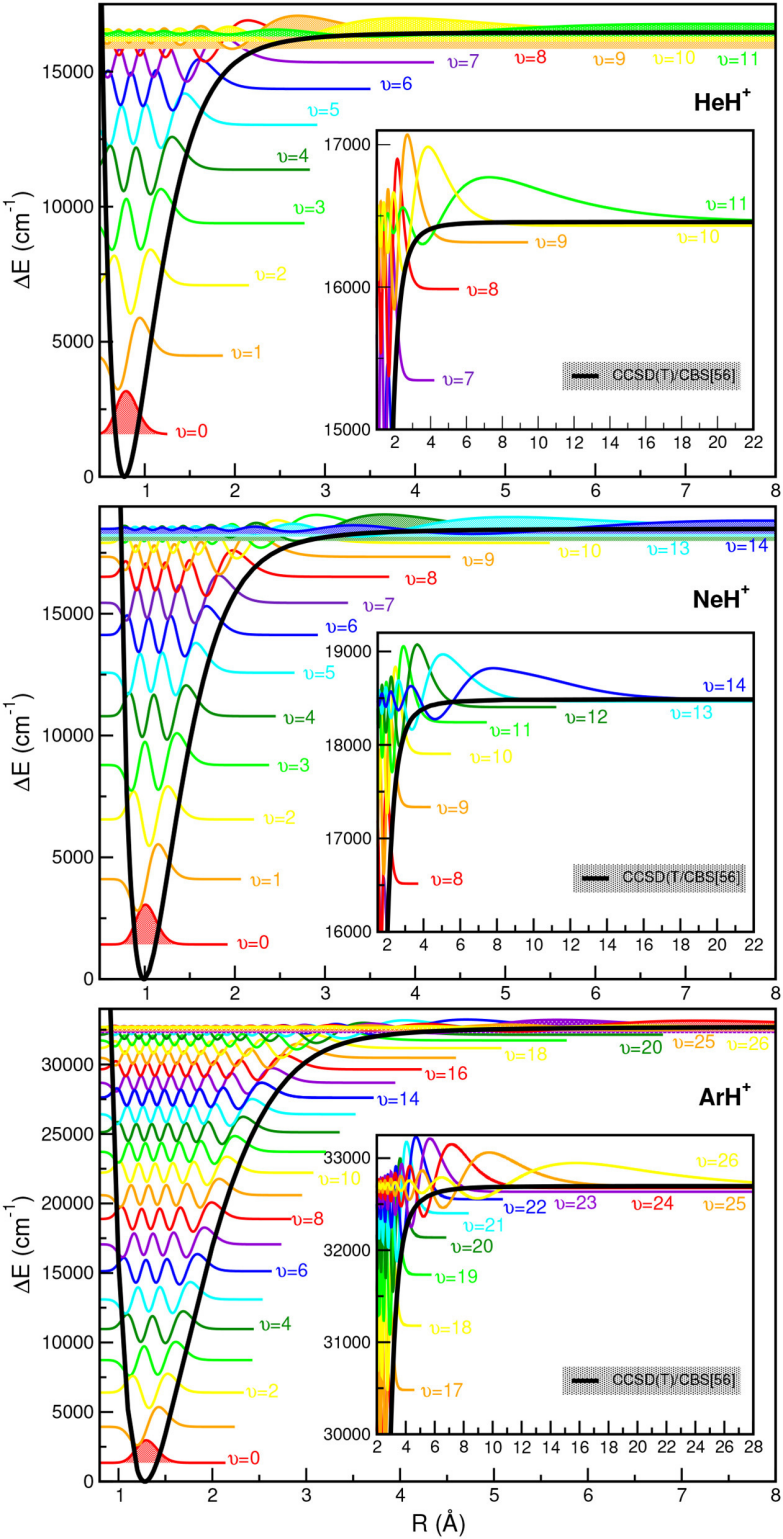
Potential energy curves of ground state NgH^+^ molecules together with the radial distributions for all calculated vibrational bound states.

In Figure 5, all eigenfunctions for the indicated NgH^+^ (Ng =^4^He, ^20^Ne, ^40^Ar from upper to lower panel, respectively) isotopes are shown together with the underlying PEC. We should note that the zero of each eigenfunction has been shifted to the energy of the corresponding υ state as given in Supplementary Tables 1–3. As expected, the eigenfunctions of the highly vibrational excited states are extended to larger *R* values, with this part of the PECs clearly affects the near dissociation quantum dynamics calculations.

In turn, in Table 4 we present comparison of υ → υ′ vibrational frequencies with the best known theoretical values (Tung et al., 2012), as well as with experimental values reported (Coxon and Hajigeorgiou, 1999) for transitions up to υ = 8 of the ^4^HeH^+^, and for the 1 → 0, 2 → 1, and 3 → 2 of ^20^NeH^+^, 1 → 0, 2 → 1 of ^20^NeD^+^, and the 1 → 0 vibrational band of ^22^NeH^+^ (Ram et al., 1985), as well as all separations (Brault and Davis, 1982) between υ = 0 up to 5 from the ^40^ArH^+^. One can see the excellent agreement achieved in the case of the ^4^HeH^+^ with differences of less than 1 cm^−1^ even for the higher vibrational bands, while such difference is also found for the lower bands of the three NeH^+^ isotopes, while in case of υ up to 5 for ^40^ArH^+^ the observed deviations are slightly higher between 2 and 5 cm^−1^. For further comparisons in Supplementary Figure 2 (see Supplementary Material), the calculated rovibrational levels are depicted for the ^4^HeH^+^ case. We should point out here that *J* > 0 could be also of interest in astrophysical applications, although we consider that their detailed investigation should be addressed in future computational spectral simulations for assigning transitions for specific isomers of the noble gas hydride cations.

**Table 4:** Vibrational υ → υ′ frequencies (in cm^−1^) of all computed bound states for the indicated NgH^+^ isotopes, and comparison with available best known or experimental values.

	^**4**^**HeH**^**+**^		^**20**^**NeH**^**+**^**(**^**20**^**NeD**^**+**^**)/**^**22**^**NeH**^**+**^		^**40**^**ArH**^**+**^	
**υ → υ′**	**From^*a*^/^*b*^**	**This work**	**From^*c*^**	**This work**	**From^*d*^**	**This work**
1 → 0	2911.0174/2910.8698	2911.425	2677.86(1984.59)/2672.50	2678.80(1985.65)/2673.45	2589.2803	2592.392
2 → 1	2604.2053/2604.1468	2604.561	2453.41(1866.36)/-	2453.37/(1865.27)/2448.98	2470.4824	2472.665
3 → 2	2295.6350/2295.5776	2296.111	2230.5	2230.44(1749.87)/2227.03	2354.5593	2357.226
4 → 3	1982.1338/1982.0808	1982.784		2007.28(1632.08)/2004.84	2241.3801	2247.186
5 → 4	1660.4510/1660.3924	1661.280		1781.92(1514.50)/1780.51	2130.7724	2135.512
6 → 5	1327.9060/1327.8469	1328.951		1551.91(1397.74)/1551.56		2025.643
7 → 6	984.4969/984.4453	985.727		1315.69(1279.01)/1316.48		1919.666
8 → 7	639.3449/640.3172	640.638		1071.45(1158.99)/1073.43		1815.963
9 → 8	327.4952	328.578		819.97(1036.13)/823.51		1711.330
10 → 9	116.2242	116.541		568.98(910.73)/573.10		1608.228
11 → 10	24.4392	24.438		337.29(782.23)/341.55		1505.493
12 → 11				162.387(650.554)/165.697		1401.692
13 → 12				61.5322(518.061)/63.331		1296.496
14 → 13				16.911(386.962)/17.597		1188.398
15 → 14						1076.222
16 → 15						958.158
17 → 16						832.442
18 → 17						697.400
19 → 18						552.743
20 → 19						402.814
21 → 20						261.787
22 → 21						151.511
23 → 22						80.784
24 → 23						39.183
25 → 24						16.050
26 → 25						4.662

a *Tung et al. (2012),*

b *Coxon and Hajigeorgiou (1999),*

c *Ram et al. (1985), and*

d *Brault and Davis (1982)*.

Finally, the calculated molecular spectroscopic constants of the present CCSD(T)/CBS[56] PECs, such as binding energies D_0_, equilibrium vibrational frequencies ω_*e*_, anharmonicity constants ω_*e*_x_*e*_, and rotational constants (B_*e*_ and B_0_), are listed in Supplementary Table 4 (see in Supplementary Material) for all NgH^+^ isotopes studied here, and compared with previous theoretical and experimental data available (Brault and Davis, 1982; Ram et al., 1985; Hirst et al., 1992; Stanke et al., 2006; Gerivani et al., 2015).

## 4. Conclusions

The present study is focused on the computational characterization of noble gas hydride NgH^+^ (Ng = He, Ne, Ar) cations, the simplest noble gas-containing molecules. Our results provide benchmark data on the underlying interactions and spectroscopic constants, determined from high-level and well-converged *ab initio* electronic structure and quantum nuclear calculations, respectively.

Two of these molecules, in particular ^4^HeH^+^ and ^36^ArH^+^/^38^ArH^+^ isotopes, have been already detected in the ISM, and has been recently extensively studied, in relation with their formation and destruction mechanisms. Here, we presented new spectroscopic data on binding energies and vibrational transitions from quantum calculations for all known stable isotopic structures in ISM and on earth of the lighter NgH^+^ molecules. These data were compared with the corresponding values reported in previous studies available, and it was found that they could serve as a benchmark for their ground electronic states.

As the accuracy issues have profound implications in developing chemoinformatics models, such reference datasets can serve to guide and cross-check computational approaches for building up predictive data-driven models for larger cationic noble gas hydrides, e.g., Ng_*n*_H^+^ clusters, and/or studying excited electronic states of these cations involved in interstellar reactions networks. In general, further difficulties may arise by increasing the complexity of the problem, in which it could challenge standard chemical assumptions. Meanwhile, it would be of interest to investigate how well these new *ab initio* potential and dipole moment curves are able to account for results in collision and photodissociation dynamics calculations of relevance in astrophysical environments, and the present work represents the first step toward this direction.

## Data Availability Statement

The original contributions presented in the study are included in the article/Supplementary Material, further inquiries can be directed to the corresponding author/s.

## Author Contributions

All authors have contributed to the work and approved it for publication.

## Conflict of Interest

The authors declare that the research was conducted in the absence of any commercial or financial relationships that could be construed as a potential conflict of interest.
