# Supplementary material for: Computational Characterization of Astrophysical Species: The Case of Noble Gas Hydride Cations
Source: Front Chem. 2021 May 11;9:664693. doi: 10.3389/fchem.2021.664693 (PMC8144312; doi:10.3389/fchem.2021.664693)
Supplement: Supplementary file 1 [file Data_Sheet_1.PDF]

Supplementary Material

1 SUPPLEMENTARY MATERIAL

1.1 Figures

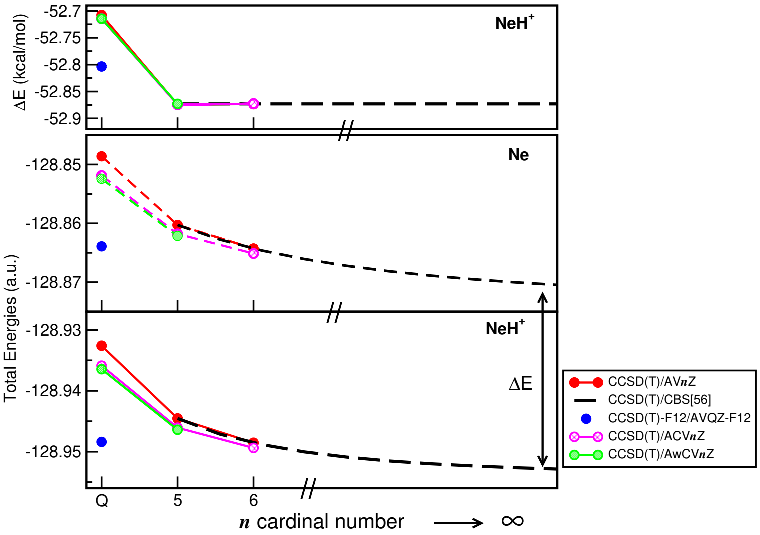

Figure S1. Convergence of total and interaction energies of NeH^+ for the indicated basis sets from CCSD(T) and CCSD(T)/CBS[56] calculations

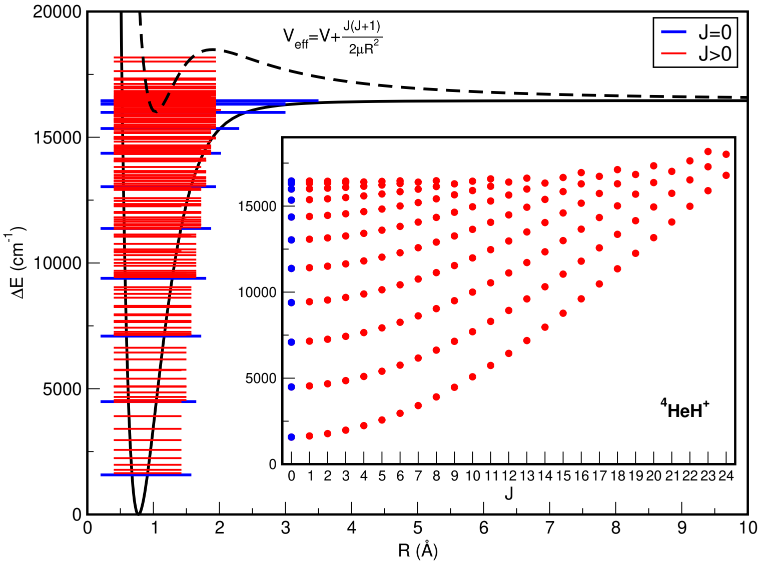

Figure S2. Rovibrational bound states of $^4HeH^+$ using the CCSD(T)/CBS[56] PEC (see solid black line). The effective potential curve for J=24 is also plotted (see dashed line).

1.2 Tables

Table S1. *

Table S1: Vibrational energies (in cm^{-1}) obtained from quantum variational bound states calculations for the indicated HeH^+ isotopes using the CCSD(T)/CBS[56] PEC.

v	^{3}**HeH**$^+$	^{4}**HeH**$^+$	^{3}**HeD**$^+$	^{4}**HeD**$^+$
0	1624.370	1574.585	1290.983	1226.761
1	4619.750	4486.010	3714.341	3537.303
2	7288.052	7090.571	5933.111	5663.428
3	9627.303	9386.682	7947.056	7605.050
4	11631.784	11369.466	9754.501	9360.923
5	13292.067	13030.746	11352.313	10928.629
6	14595.964	14359.697	12735.856	12304.496
7	15532.369	15345.424	13899.307	13483.830
8	16104.776	15986.062	14836.311	14461.182
9	16366.976	16314.639	15542.194	15231.609
10	16445.213	16431.180	16019.530	15793.467
11	16456.824	16455.618	16290.680	16155.403
12			16410.779	16348.396
13			16449.625	16428.888
14			16456.733	16453.186

Table S2. *

Table S2: Vibrational energies (in cm^{-1}) obtained from quantum variational bound states calculations for the indicated NeH^+ isotopes using the CCSD(T)/CBS[56] PEC.

v	^{20}**NeH**$^+$	^{21}**NeH**$^+$	^{22}**NeH**$^+$	^{20}**NeD**$^+$	^{21}**NeD**$^+$	^{22}**NeD**$^+$
0	1425.154	1423.549	1422.094	1036.558	1034.317	1032.284
1	4103.955	4099.543	4095.543	3022.209	3015.894	3010.162
2	6557.321	6550.608	6544.522	4887.480	4877.596	4868.623
3	8787.756	8779.2603	8771.555	6637.346	6624.408	6612.662
4	10795.037	10785.264	10776.399	8269.423	8253.946	8239.891
5	12576.959	12566.449	12556.913	9783.919	9766.400	9750.486
6	14128.871	14118.176	14108.470	11181.655	11162.630	11145.345
7	15444.557	15434.284	15424.954	12460.661	12440.664	12422.489
8	16516.002	16506.774	16498.388	13619.652	13599.233	13580.665
9	17335.973	17328.415	17321.537	14655.782	14635.529	14617.103
10	17904.951	17899.558	17894.639	15566.511	15547.044	15529.320
11	18242.245	18239.084	18236.193	16348.736	16330.673	16314.209
12	18404.632	18403.203	18401.890	16999.290	16983.292	16968.688
13	18466.1642	18465.675	18465.221	17517.351	17503.954	17491.698
14	18483.075	18482.943	18482.818	17904.313	17894.001	17884.535
15				18168.793	18161.633	18155.036
16				18332.185	18327.799	18323.729
17				18419.843	18417.465	18415.254
18				18462.4001	18461.332	18460.323
19				18478.707	18478.271	18477.867
20				18484.526	18484.456	18484.390

Table S3. *

Table S3: Vibrational energies (in cm^{-1}) obtained from quantum variational bound states calculations for the indicated ArH^+ isotopes using the CCSD(T)/CBS[56] PEC.

v	^{36}**ArH**$^+$	^{38}**ArH**$^+$	^{40}**ArH**$^+$	^{36}**ArD**$^+$	^{38}**ArD**$^+$	^{40}**ArD**$^+$
0	1345.161	1344.207	1343.346	967.142	965.803	964.594
1	3940.927	3938.200	3935.737	2852.580	2848.698	2845.191
2	6416.645	6412.313	6408.403	4676.049	4669.797	4664.148
3	8776.629	8770.848	8765.629	6438.272	6429.817	6422.178
4	11026.278	11019.202	11012.815	8141.214	8130.706	8121.213
5	13163.945	13155.737	13148.327	9787.124	9774.717	9763.509
6	15191.470	15182.273	15173.969	11375.266	11361.127	11348.351
7	17112.758	17102.709	17093.636	12906.059	12890.342	12876.140
8	18930.058	18919.307	18909.599	14380.196	14363.047	14347.550
9	20642.443	20631.138	20620.929	15799.237	15780.790	15764.120
10	22251.467	22239.744	22229.157	17164.505	17144.900	17127.182
11	23757.461	23745.475	23734.651	18475.909	18455.306	18436.683
12	25159.356	25147.264	25136.343	19733.198	19711.741	19692.346
13	26455.729	26443.703	26432.838	20937.094	20914.919	20894.873
14	27643.645	27631.873	27621.236	22088.134	22065.385	22044.819
15	28718.978	28707.675	28697.458	23186.080	23162.911	23141.963
16	29675.782	29665.192	29655.617	24230.747	24207.311	24186.117
17	30506.333	30496.738	30488.059	25221.781	25198.238	25176.945
18	31201.235	31192.955	31185.458	26158.508	26135.031	26113.795
19	31750.870	31744.225	31738.201	27039.949	27016.722	26995.706
20	32150.167	32145.371	32141.015	27864.790	27842.012	27821.396
21	32408.625	32405.576	32402.801	28631.253	28609.143	28589.125
22	32557.663	32555.910	32554.312	29337.118	29315.918	29296.715
23	32636.879	32635.947	32635.096	29979.691	29959.669	29941.523
24	32675.110	32674.676	32674.279	30555.790	30537.243	30520.419
25	32690.634	32690.475	32690.329	31061.803	31045.054	31029.845
26	32695.051	32695.020	32694.992	31494.050	31479.427	31466.128
27				31849.589	31837.390	31826.273
28				32127.639	32118.037	32109.262
29				32332.135	32325.059	32318.575

Table S4. *

Table S4:Computed spectroscopic constants (in cm^{-1}) of the indicated ground state RgH^+ isotopes.

	$\mathbf{D}_0$	**ZPE**	ω_e	$\omega_e\mathbf{x}_e$	$\mathbf{B}_e$	$\mathbf{B}_0$
^{3}**HeH**$^+$	14832.5842	1624.3698	2668.3017	163.5392	37.2032	35.7113
^{4}**HeH**$^+$	14882.3693	1574.5847	2604.5613	153.4318	34.9096	33.5559
Expt. ^{a}	14889.5636	1566.6764	2604.1468	153.4065	34.9250	33.5586
^{3}**HeD**$^+$	15165.9707	1290.9833	2218.7699	102.2937	23.2889	22.5536
^{4}**HeD**$^+$	15230.1928	1226.7612	2126.1247	92.2087	20.9952	20.3663
Expt. ^{a}	15229.6765	1226.5635	2126.1792	92.1522	20.9853	20.3495
^{20}**NeH**$^+$	17060.8673	1425.1537	2904.2365	112.7177	17.8795	17.3246
Expt. ^{b}	-	-	2903.751	113.3581	17.8847	17.3381
Theor. ^{c}	17026.41	-	2892.077	109.885	17.7171	17.1858
Theor. ^{d}	17023.98	1425.02	2896.62	111.30	17.828	17.3362
^{21}**NeH**$^+$	17062.4721	1423.5489	2900.9229	112.4625	17.8377	17.2848
^{22}**NeH**$^+$	17063.9270	1422.0940	2897.919	112.2350	17.8019	17.2507
^{20}**NeD**$^+$	17449.4631	1036.5579	2106.0313	60.1900	9.3867	9.1744
^{21}**NeD**$^+$	17451.7038	1034.3172	2101.4520	59.9375	9.3450	9.0990
^{22}**NeD**$^+$	17453.7374	1032.2836	2097.2964	59.7088	9.3092	9.1340
^{36}**ArH**$^+$	31350.3268	1345.1612	2715.8147	60.0242	10.4784	10.2832
^{38}**ArH**$^+$	31351.2810	1344.2070	2713.8790	59.9395	10.4635	10.2687
^{40}**ArH**$^+$	31352.1424	1343.3456	2712.1178	59.8631	10.4497	10.2552
Expt. ^{e}	-	-	2710.9199	61.6377	10.4613	10.2726
Theor. ^{c}	31388.20	-	2686.806	55.765	10.3054	10.1834
^{36}**ArD**$^+$	31728.3462	967.1428	1947.4070	30.9844	5.3929	5.4086
^{38}**ArD**$^+$	31729.6850	965.8030	1944.6915	30.8982	5.3779	5.3052
^{40}**ArD**$^+$	31730.8944	964.5936	1942.2387	30.8205	5.3641	5.2916

^{a} Coxon and Hajigeorgiou (1999), ^{b} Ram et al. (1985), ^{c} Hirst et al. (1992), ^{d} Gerivani et al. (2015), ^{e} Brault and Davis (1982).

REFERENCES

Brault, J. W. and Davis, S. P. (1982). Fundamental vibration-rotation bands and molecular constants for the ArH ground state $^1\Sigma^+$. *Phys. Scr.* 25, 268–271. doi:10.1088/0031-8949/25/2/004

Coxon, J. and Hajigeorgiou, P. (1999). Experimental Born–Oppenheimer potential for the $X^1\Sigma^+$ ground state of HeH^+: Comparison with the ab initio potential. *J. Mol. Spectrosc.* 193, 306–318. doi:10.1006/jmsp.1998.7740

Gerivani, B., Shayesteh, A., and Maghari, A. (2015). Ab initio potential energy curves and transition dipole moments for the low-lying electronic states of NeH^+. *Comput. Theor. Chem.* 1070, 82–87. doi:10.1016/j.comptc.2015.07.027

Hirst, D., Guest, M., and Rendell, A. (1992). Ab initio potential-energy curves for the molecular ions NeH^+ and ArH^+. *Mol. Phys.* 77, 279–290. doi:10.1080/00268979200102441

Ram, R., Bernath, P., and Brault, J. (1985). Fourier transform emission spectroscopy of NeH^+. *J. Mol. Spectrosc.* 113, 451–457. doi:10.1016/0022-2852(85)90281-4
